# Medical expenses in treating acute esophageal variceal bleeding: A 15-year nationwide population-based cohort study

**DOI:** 10.1097/MD.0000000000004215

**Published:** 2016-07-18

**Authors:** Chueh-Ling Liu, Cheng-Kun Wu, Hon-Yi Shi, Wei-Chen Tai, Chih-Ming Liang, Shih-Cheng Yang, Keng-Liang Wu, Yi-Chun Chiu, Seng-Kee Chuah

**Affiliations:** a Division of Hepatogastroenterology, Department of Internal Medicine, Kaohsiung Chang Gung Memorial Hospital; b Department of Healthcare Administration and Medical Informatics, Kaohsiung Medical University, Kaohsiung; c Chang Gung University, College of Medicine, Taipei, Taiwan.

**Keywords:** esophageal variceal bleeding, length of hospital stay, medical expenses, mortality, prevalence

## Abstract

Acute variceal bleeding in patients with cirrhosis is related to high mortality and medical expenses. The purpose of present studies was to analyze the medical expenses in treating acute esophageal variceal bleeding among patients with cirrhosis and potential influencing clinical factors.

A total of 151,863 patients with cirrhosis with International Classification of Diseases-9 codes 456.0 and 456.20 were analyzed from the Taiwan National Health Insurance Research Database from January 1, 1996 to December 31, 2010. Time intervals were divided into three phases for analysis as T1 (1996–2000), T2 (2001–2005), and T3 (2006–2010). The endpoints were prevalence, length of hospital stay, medical expenses, and mortality rate.

Our results showed that more patients were <65 years (75.6%) and of male sex (78.5%). Patients were mostly from teaching hospitals (90.8%) with high hospital volume (50.9%) and high doctor service load (51.1%). The prevalence of acute esophageal variceal bleeding and mean length of hospital stay decreased over the years (*P* < 0.001), but the overall medical expenses increased (*P* < 0.001). Multiple regression analysis showed that older age, female sex, Charlson comorbidity index (CCI) score >1, patients from teaching hospitals, and medium to high or very high patient numbers were independent factors for longer hospital stay and higher medical expenses. Aged patients, female sex, increased CCI score, and low doctor service volume were independent factors for both in-hospital and 5-year mortality. Patients from teaching hospitals and medium to high or very high service volume hospitals were independent factors for in-hospital mortality, but not 5-year mortality.

Medical expenses in treating acute esophageal variceal bleeding increased despite the decreased prevalence rate and length of hospital stay in Taiwan. Aged patients, female sex, patients with increased CCI score from teaching hospitals, and medium to high or very high patient numbers were the independent factors for increased medical expenses.

## 1 Introduction

Approximately, one-third of patients with cirrhosis develop variceal bleeding, with 70% recurrence rates and 20% to 50% might encounter death.^[1–4]^ One-third of patients had episodes of rebleeding within 6 weeks and two thirds of the patients had them within 1 year.^[5]^ The bleeding events were related to the subsequent medical expenses and high mortality. With the recent advances in the strategies for primary prophylaxis, decreased prevalence of acute esophageal variceal bleeding can be expected. These include treatments such as endoscopic variceal ligation and antibiotic prophylaxis, causing the decrease in the mortality rate over the past two decades in western countries.^[6–8]^ In contrast, a trend of increasing medical expenses was observed for acute esophageal variceal bleeding, despite advances in treatment.^[9]^ To our knowledge, most studies in the literature had short-term follow up (<5 years) data, and the long-term follow-up data were never discussed.

Hence, we conducted a nationwide 15-year population-based study to analyze the medical expenses and mortality in treating acute esophageal variceal bleeding and their potential influencing clinical factors among patients with cirrhosis.

## 2 Patients and methods

### 2.1 Data source

**Figure 1:**
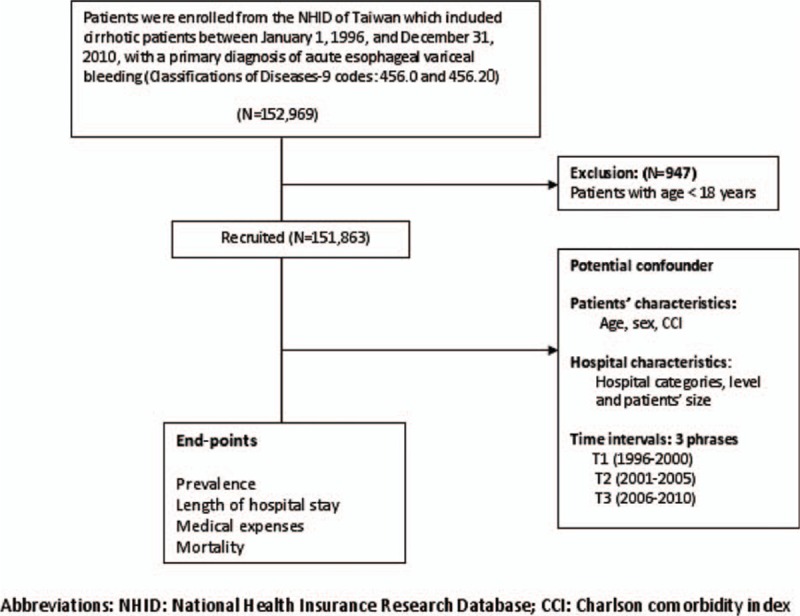
Schematic flowchart of the study design.

Figure 1 shows the schematic flowchart of the study design. The database used in this study included 151,863 randomly selected subjects from the 1996 to 2010 Taiwan National Health Insurance Research Database (NHIRD), which was developed for research purposes. The NHIRD is a research database developed at the National Health Research Institute, with linked data from the demographic and enrollment records, hospital claims, ambulatory care visits, and pharmacy-dispensing claims from hospitals, outpatient clinics, and community pharmacies. Our source population comprised all beneficiaries who were at least 18 years old from the longitudinal Health Insurance Database 1996. No statistically significant differences were found in age, sex, and average insured payroll-related amount between the sample group and all enrollees. This study was approved by both the Institutional Review Board and Ethics Committee of Chang Gung Memorial Hospital, Taiwan (IRB104–9833B). The Ethics Committee waived the requirement for informed consent, and each patient's medical records were anonymized and de-identified before access.

### 2.2 Hypothesis

We hypothesized that prevalence of patients with cirrhosis complicated with esophageal variceal bleeding and the length of hospital stay decreased over time in Taiwan, but the medical expenses in this study population was increasing.

### 2.3 Study aims

The purpose of the present study was to analyze the prevalence, length of hospital stay, medical expenses, and mortality rate as well as and their potential influencing clinical factors among patients with cirrhosis with acute esophageal variceal bleeding.

### 2.4 Study population

Patients were enrolled from the NHIRD of Taiwan, which included patients with cirrhosis between January 1, 1996 and December 31, 2010. To reduce heterogeneity, we used the International Classification of Diseases (ICD)-9 codes 571 in order to focus on patients with chronic liver disease and cirrhosis of various etiologies (including alcoholism, chronic hepatitis B/C, autoimmune disease, biliary cirrhosis, etc.). Subsequently, we used the diagnosis of acute esophageal variceal bleeding (ICD-9 codes 456.0 and 456.20) for further investigation. The ICD-9 code 456.0 defines diagnosis of esophageal varices with bleeding. The ICD-9 code 456.20 defines esophageal varices in diseases classified elsewhere with bleeding from either cirrhosis of the liver or portal hypertension. By using ICD-9 code 571 in the primary selection, we could confirm that patients enrolled in this study had a diagnosis of esophageal variceal bleeding because of liver cirrhosis or portal hypertension of liver etiology (most were still caused by cirrhosis).

We analyzed data, which included patient characteristics such as age, sex, Charlson comorbidity index (CCI), hospital characteristics such as hospital categories, level, and size. Time intervals were divided into three phases for analysis as T1 (1996–2000), T2 (2001–2005), and T3 (2006–2010). The endpoints were prevalence, medical expenses, and mortality rate. The CCI index was designed to objectively measure the comorbidity burden and establish through estimation 1-year mortality rates in hospitalized medical patients.^[10,11]^ The index scores diseases on a 6-points scale. For example, 1 point was assigned to patients with conditions such as myocardial infarction, congestive heart failure, peripheral arterial disease, cerebrovascular disease, dementia, chronic pulmonary disease, connective tissue disease, ulcer disease, mild liver disease, and diabetes without organ damage. A score of 2 was assigned to patients with diabetes with organ damage, hemiplegia, severe renal disease, and nonmetastatic cancer. In cases where the patient had severe liver disease, a score of 3 and 6 points were assigned to those who had metastatic cancer and human immunodeficiency virus infection. Finally, we calculated the total points for each patient and considered the points as the comorbidity burden.

### 2.5 Statistical analysis

All results were expressed as means ± standard deviations and frequencies or percentages for continuous and categorical data, respectively. Distributions of continuous variables were analyzed by using the Mantel–Haenszel chi square test for trend. Kaplan–Meier and Cox regression analyses were used for survival rate analysis. Variables were analyzed by using multiple regression model to determine the independent predictive factors during trend forecast analysis. Only the variables significant in the univariate analysis were analyzed in the multivariate analysis. The results were expressed as odds ratios (OR) with 95% confidence intervals. Statistical significance was set at *P* < 0.05. All analyses were performed by using SPSS ver. 19 (SPSS, Inc, Chicago, IL).

## 3 Results

### 3.1 Basic demographic data of patients with cirrhosis with acute esophageal variceal bleeding

A total of 152,969 patients with cirrhosis who had esophageal variceal bleeding were enrolled from the NHIRD between January 1, 1996 and December 31, 2010 (ICD-9 codes 456.0 and 456.20). After we excluded 947 patients aged <18 years, 151,863 patients were eventually enrolled for the analysis. As summarized in Table 1, 75.6% of patients were <65 years and 78.5% were of male sex. There were 68.9% of patients with low CCI score (0–1). Patients were mostly from teaching hospitals (90.8%), from either the medical center or district hospital (81.5%), with high hospital volume (50.9%) and high doctor services (51.1%). The mean length of hospital stay was 9.8 ± 8.3 days.

**Table 1:**
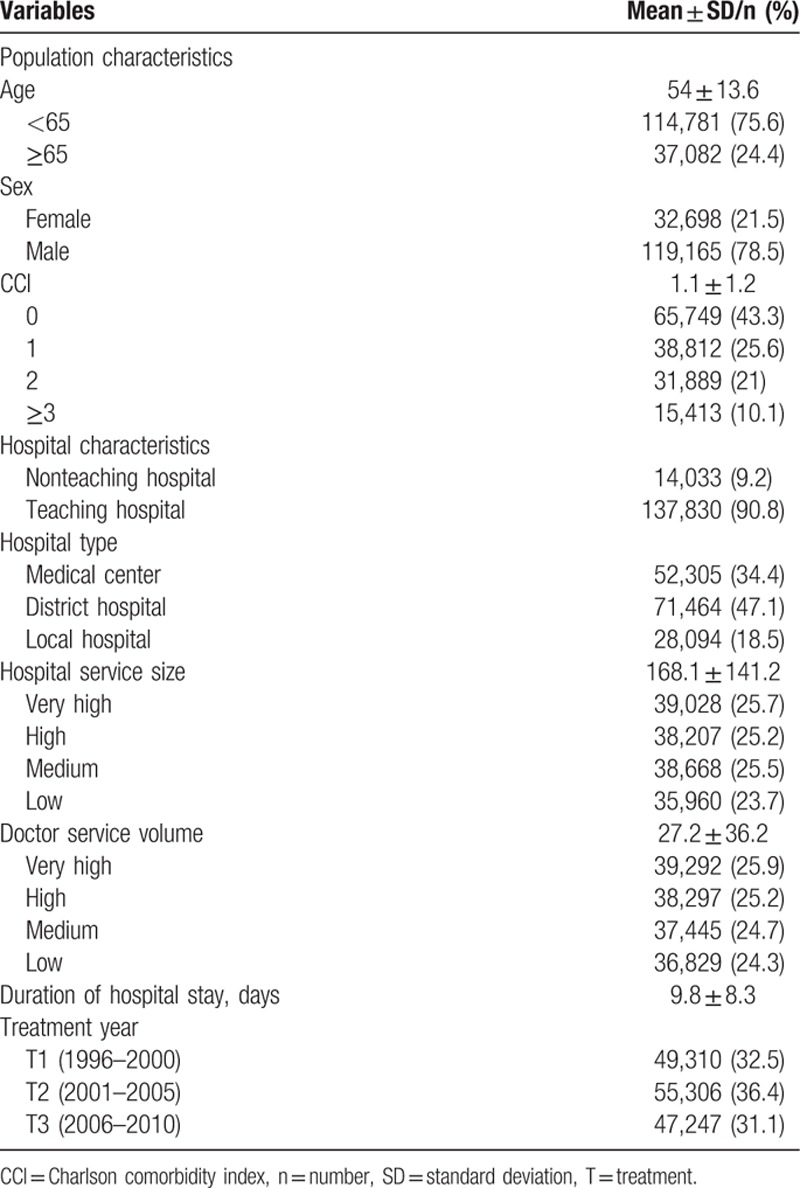
Basic demographic data of patients with cirrhosis with acute esophageal variceal bleeding.

**Figure 2:**
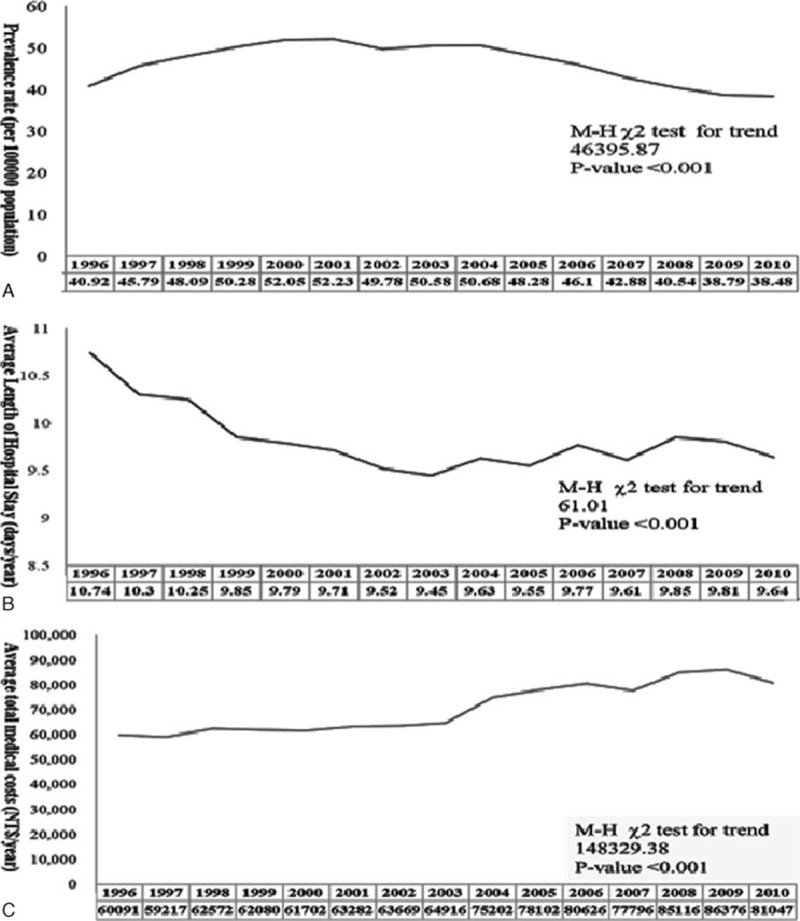
Prevalence, length of hospital stay, and medical expenses in acute variceal bleeding: (A) prevalence rate; (B): length of hospital stay; (C): medical expenses.

**Figure 3:**
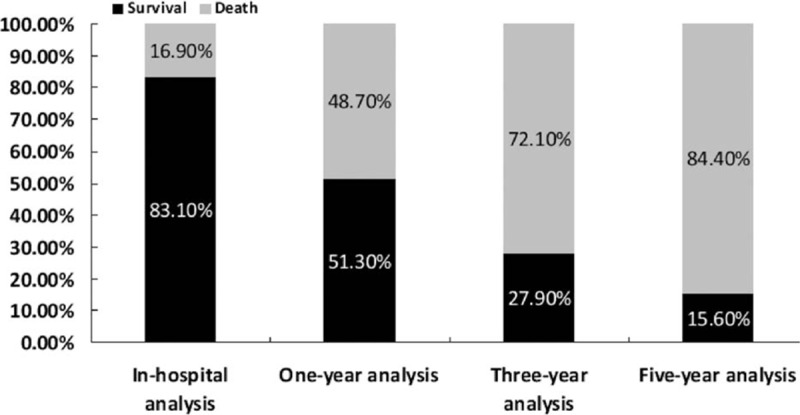
Mortality rates of acute esophageal variceal bleeding.

**Figure 4:**
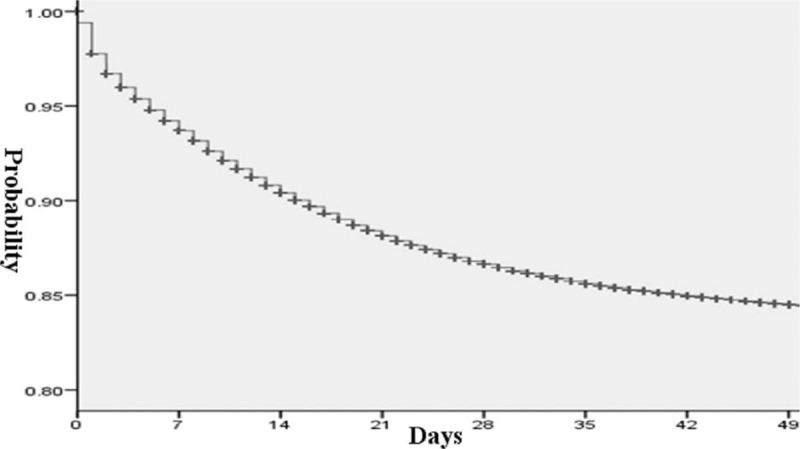
Kaplan–Meier survival curves for in hospital analysis.

**Figure 5:**
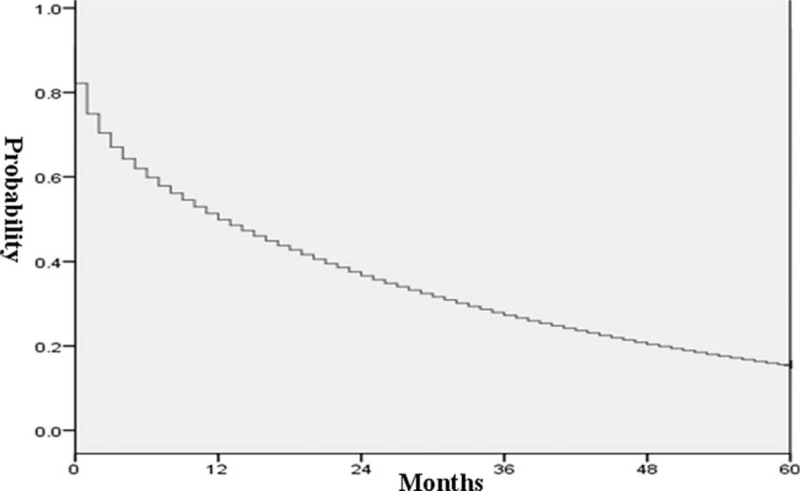
Kaplan–Meier survival curves for 5-year analysis.

The results showed that the prevalence of acute esophageal variceal bleeding and the mean length of hospital stay had decreased significantly over the years, as shown in Fig. 2A and B (*P* < 0.001 by Mantel–Haenszel χ^2^ test for trend). In contrast, the medical expenses increased in this population as shown in Fig. 1C (*P* < 0.001). The in-hospital mortality rate was estimated to be 16.9%, but increased subsequently to 48.7%, 72.1%, and 84.4% for 1-, 3-, and 5-year analyses (Fig. 3). The median in-hospital survival time was 13.31 days and the median 5-year survival time was 14.54 months (Figs. 4 and 5). The total amount of medical expenses is summarized in Table 2.

**Table 2:**
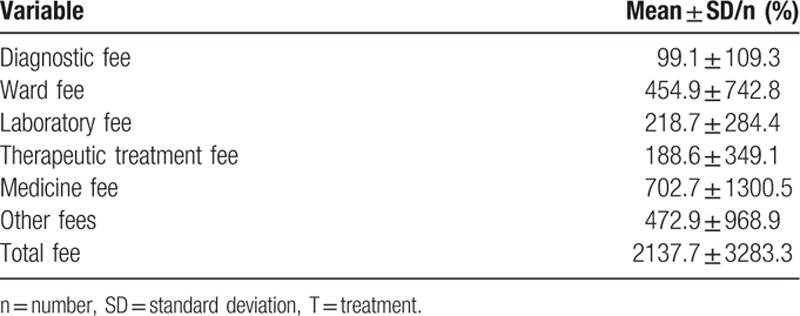
Medical expenses of patients with cirrhosis with acute esophageal variceal bleeding (in US dollars).

### 3.2 Trend analysis of demographic and institutional characteristics of patients with cirrhosis for acute esophageal variceal bleeding based on time intervals

Significantly more aged patients (≥65 years), patients with a CCI score of 2, and patients from teaching hospitals such as the medical center or district hospital with very high hospital service volume and low doctor service volume were observed in the T3 group compared with the T1 group. In contrast, significantly less patients aged <65 years, CCI score of 0, and from nonteaching hospitals such as local hospitals with very high doctor service volume were observed in the T3 group compared with the T1 group (Table 3).

**Table 3:**
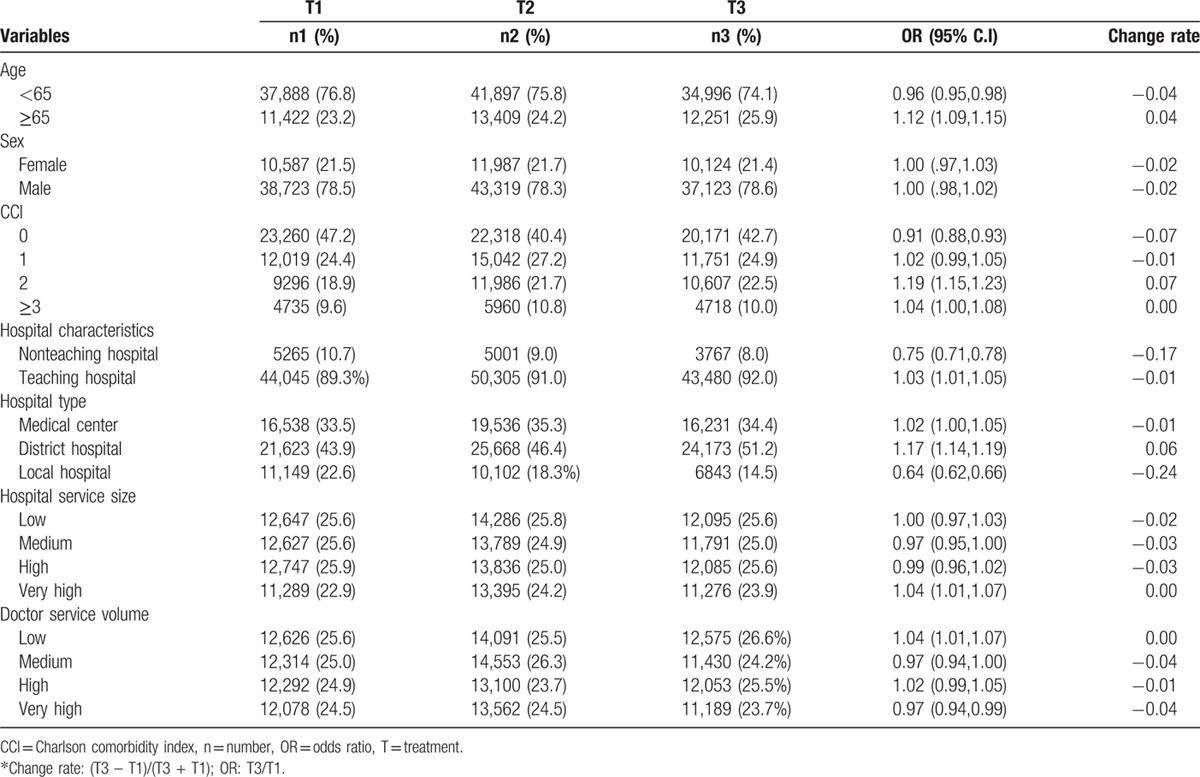
Trend analysis of demographic and institutional characteristics of patients with cirrhosis with acute esophageal variceal bleeding.

### 3.3 Factors influencing the length of hospital stay and medical expenses of patients with cirrhosis for acute esophageal variceal bleeding

Multiple regression analysis showed that older age, female sex, CCI score >1, patients from teaching hospitals, and characterized as having medium to high or very high patient numbers were the independent factors for longer hospital stay. In contrast, female sex, patients from the area or local hospital, and high or very high doctor service volume were negative factors for longer hospital stay (Table 4).

**Table 4:**
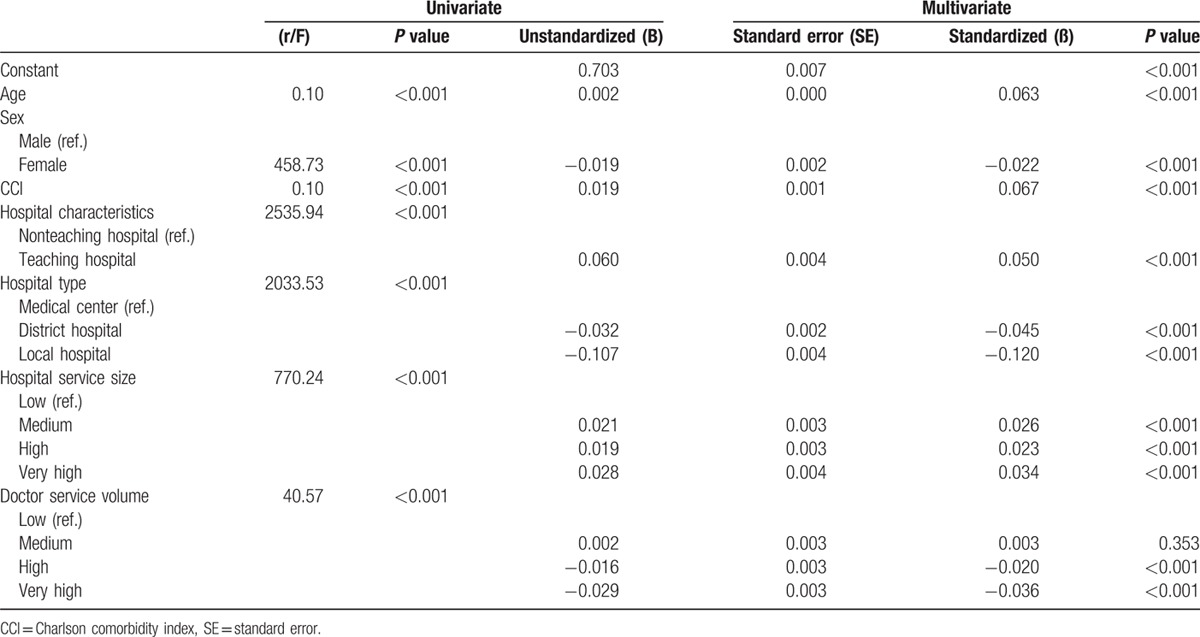
Factors influencing length of hospital stay of patients with cirrhosis for acute esophageal variceal bleeding.

Simultaneously, factors associated with higher medical expenses were older age, female sex, CCI score >1, patients from teaching hospitals, and characterized as having medium to high or very high patient numbers were the independent factors for increased medical expenses. Longer length of hospital stay also indicated higher medical expenses. Factors including patients from local hospitals with medium to high or very high doctor service volume were associated with lower medical expenses (Table 5).

**Table 5:**
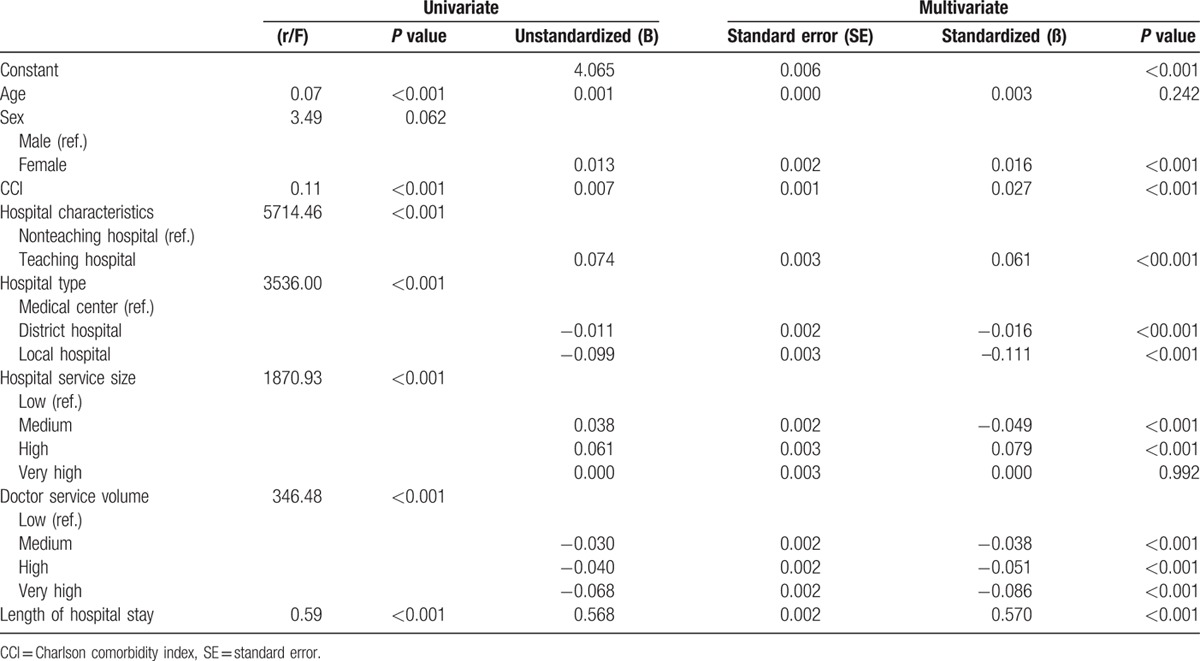
Factors influencing medical expenses of patients with cirrhosis for acute esophageal variceal bleeding.

### 3.4 Factors influencing in-hospital and 5-year mortality rates of patients with cirrhosis for acute esophageal variceal bleeding

As shown in Tables 6 and 7, aged patients, female sex, CCI score >1, and hospitals with medium to high or very high doctor service volume were independent factors for both in-hospital and 5-year mortality rates. For hospital characteristics and service size, patients from teaching hospitals, and with medium to high or very high doctor service volume were independent factors for in-hospital mortality rate, but not in 5-year mortality rate.

**Table 6:**
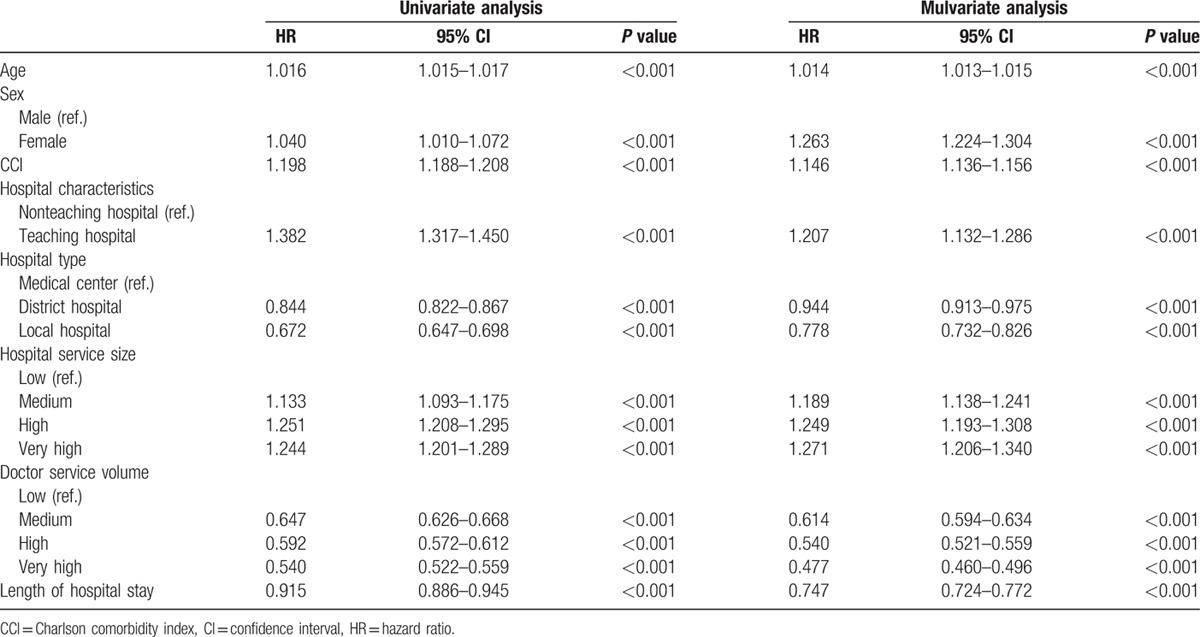
Factors influencing in-hospital mortality rate of patients with cirrhosis for acute esophageal variceal bleeding.

**Table 7:**
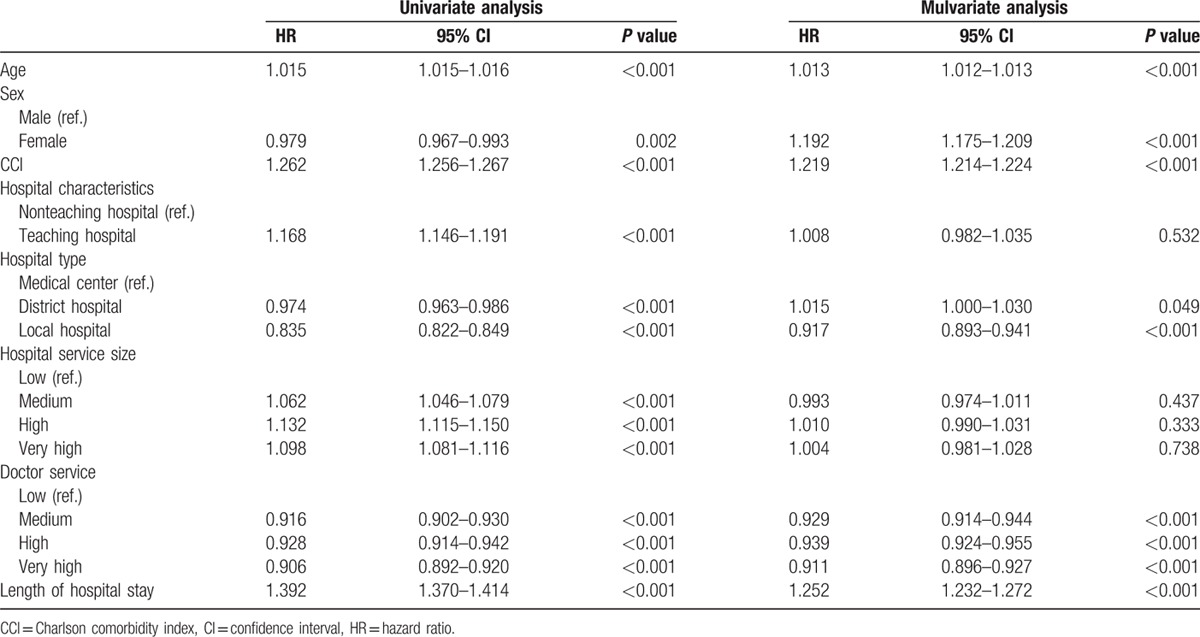
Factors influencing the 5-year mortality rate of patients with cirrhosis for acute esophageal variceal bleeding.

## 4 Discussion

In this 15-year nationwide population-based cohort study, a significantly decreased prevalence rate of acute esophageal variceal bleeding was observed. The decreased prevalence rate is reasonable because the improvement in prophylaxis strategy and advances in endoscopic variceal ligation had decreased the risk of acute bleeding episode and mortality rate.^[6,12–14]^ Besides, recent advances in the treatment of underlying disease, such as hepatitis B and C, prevent the development into liver cirrhosis and subsequent portal hypertension, which lead to the complication of varices.^[15–17]^ These antiviral therapies can even regress pre-existing esophageal varices despite the fact that the clinical benefit of treating patients with hepatitis C with late-stage disease is still poorly understood and must be investigated.^[18]^ Approximately a decade ago, Liaw et al^[19]^ proved that continuous treatment with lamivudine delays clinical progression in patients with chronic hepatitis B and advanced fibrosis or cirrhosis by significantly reducing the incidence of hepatic decompensation and hepatocellular carcinoma risk. Since then, increasing evidence supports the concept that liver cirrhosis is reversible in nucleos(t)ide analog (NucSaurabh)-treated patients with hepatitis B with maintained or sustained hepatitis B virus suppression and resolved hepatitis activity.^[20]^ In addition, the adherence to the consensus guidelines is an important issue in portal hypertension.^[21–25]^ Owing to the adherence to the guidelines for primary and secondary prophylaxes in treating varices, the hospitalization rate for bleeding varices has been decreasing in America.^[26]^ The present study observed that the decreased length of hospital stay when treating variceal bleeding implied the improvement of clinical care in Taiwan, such as vasoconstrictor prescription (e.g., terlipressin), endoscopic hemostasis, and adherence to the indication guidelines for prophylactic antibiotics.

In contrast, the present study also observed the increase in medical expenses, which was similar to that in the previous study of Viviane and Alan.^[9]^ We also found that aged patients and CCI score >1 were independent factors for both increase of length of hospital stay and medical expenses. It is rational because comorbidity is an important prognostic factor for patients with cirrhosis.^[11]^ Apart from acute esophageal bleeding, this patient cohort might have had multiple comorbidities such as heart, lung, and vascular problems, which needed more intensive medical care. This may explain why medical expenses increased in the present study.

The issue regarding hospital quality care might be reflected by the hospital volume might also influence the medical expenses. Myers et al^[27]^ showed that higher hospital volume was associated with longer hospital stay and increased charges, but no such volume–outcome relationship was found in Dy's study.^[28]^ In the present study, it was interesting to note that patients from teaching hospitals, characterized as having medium to high or very high patient numbers, were associated with longer hospital stay and higher medical expenses. In contrast, high or very high doctor service volume was associated with shorter length of hospital stay and lower medical expenses, which implied that experienced physicians, instead of hospital volume, might be responsible for determining the outcomes and the subsequent medical expenses of acute variceal bleeding.

The overall in-hospital mortality rate in the present study was 16.9%, which was similar to previous studies, ranging from 11.8% to 18%.^[6,8]^ Our 5-year result was also similar to that of Stokkel's study.^[7]^ The main reason behind the higher mortality rates could be because of factors such as aging and increased CCI score. Despite the fact that improvements in treatment had decreased the risk of mortality in acute variceal bleeding, aging process was accompanied by other comorbid diseases, leading to inevitable death in this patient group.

Regarding the issue of hospital categories and service size, previous studies showed that hospital volume was not an independent predictor for in-hospital mortality rate.^[27,29]^ The present study also observed that patients from teaching hospitals and medium to high or very high service volume were independent factors for in-hospital mortality rate, whereas no such relation was found with respect to 5-year mortality rate. This was because patients in Taiwan were allowed to seek for medical help from any hospital category as per their wish, based on the National Health Insurance System regulation. Therefore, it was not surprising that only some selected patients with relatively mild illness might seek medical help in a district or local hospital. The bottom line is most patients with cirrhosis who had hematemesis or melena choose to go to the medical centers for treatment. More patients with severe illness that resulted in higher in-hospital mortality rate were rational in teaching hospitals loaded by high or very high service volume.

Once patients survived from acute esophageal variceal bleeding, old age and CCI scores became the important factors for 5-year mortality rate. High surgeon volume associated with reduction of surgical mortality rate had been reported.^[30]^ In fact this was similar to the observation in the present study that high or very high doctor service volume had lower in-hospital and 5-year mortality rates particularly in the medical centers. This was probably because of the availability of more specialized physicians who are skillful in endoscopic hemostasis and could provide better care for patients with bleeding.

This study has several limitations. First, the lack of clinical information such as hemodynamics, laboratory data, and *Child*–*Pugh classification* did not allow us to perform more detailed regression analysis. Second, we could not gather information of patients’ own expenses from the NHIRD of Taiwan. For instance, endoscopic esophageal varices could be treated by using multiple ligators. However, this device was not covered by the National Health Insurance Company and patients needed to pay their own expenses when the device was first available in Taiwan. It was only until 8 years prior that the ligators were covered by the National health Insurance Company, which was approximately the T3 interval in the present study (2006–2010). Therefore, we might have missed some of these patients’ information during the analysis process. Third, this study did not include any information of medications used. The bottom line is, each kind of prescribed medication to these patients might have different therapeutic effect and could possibly influence the mortality rate. Lastly, this database did not contain information on perceived health status (such as quality of life questionnaire).

In conclusion, medical expenses in treating acute esophageal variceal bleeding increased despite the decreased prevalence rate and length of hospital stay in Taiwan. Aged patients, increased CCI score, and low doctor service volume were the independent factors.
